# Dose–Response Associations Between Daily Step Count, Cardiorespiratory Fitness, and Symptoms of Depression, Anxiety, and Stress in University Students

**DOI:** 10.3390/jcm15093191

**Published:** 2026-04-22

**Authors:** Andrés Godoy-Cumillaf, Paola Fuentes-Merino, Josivaldo de Souza-Lima, Maribel Parra-Saldias, Daniel Duclos-Bastias, Claudio Farias-Valenzuela, Eugenio Merellano-Navarro, José Bruneau-Chávez, Eva Rodríguez-Gutiérrez

**Affiliations:** 1 Grupo de Investigación en Educación Física, Salud y Calidad de Vida (EFISAL), Facultad de Educación, Universidad Autónoma de Chile, Temuco 4780000, Chile; andres.godoy@uautonoma.cl (A.G.-C.); paola.fuentes@uautonoma.cl (P.F.-M.); 2 Facultad de Educación y Ciencias Sociales, Instituto del Deporte y Bienestar, Universidad Andres Bello, Las Condes, Santiago 7550000, Chile; josivaldo.desouza@unab.cl; 3 Departamento de Educación Física, Deporte y Recreación, Universidad de Atacama, Copiapó 1530000, Chile; maribel.parra@uda.cl; 4 iGEO, Escuela de Educación Física, Facultad de Filosofía y Educación, Pontificia Universidad Católica de Valparaíso, Valparaíso 2340025, Chile; daniel.duclos@pucv.cl; 5 METIS Research Lab, Facultad de Negocios y Tecnología, Universidad Alfonso X el Sabio (UAX), 28691 Madrid, Spain; 6 Escuela de Ciencias de la Actividad Física, Universidad de Las Américas, Santiago 1530000, Chile; claudio.farias.valenzuela@udla.cl; 7 Department of Physical Activity Sciences, Faculty of Education Sciences, Universidad Católica del Maule, Talca 3530000, Chile; emerellano@ucm.cl; 8 Departamento de Educación Física, Deportes y Recreación, Universidad de la Frontera, Temuco 4811230, Chile; jose.bruneau@ufrontera.cl; 9 Investigación en Estudios Sociosanitarios, Instituto de Investigación Sanitaria de Castilla-La Mancha (IDISCAM), 45071 Toledo, Spain; 10 Centro de Estudios Sociosanitarios, Universidad de Castilla-La Mancha, 61071 Cuenca, Spain

**Keywords:** mental health, physical fitness, physical activity, pedometer

## Abstract

**Background/Objectives**: University life is often accompanied by unhealthy lifestyle behaviors, reduced physical activity, lower fitness levels, and a high prevalence of mental health symptoms. Daily step count has emerged as a practical indicator of habitual physical activity; however, evidence on its association with cardiorespiratory fitness and symptoms of depression, anxiety, and stress in university students remains limited. Therefore, this study examined the association of daily step count with cardiorespiratory fitness and symptoms of depression, anxiety, and stress in university students. **Methods**: This cross-sectional association study included a convenience sample of 120 students aged 18 to 25 years from a single university. Daily step count was assessed over seven consecutive days using a Xiaomi Mi Band 9. Cardiorespiratory fitness was evaluated with the 20 m shuttle run test, and symptoms of depression, anxiety, and stress were measured using the Depression, Anxiety and Stress Scale-21 Items (DASS-21). Partial correlations, ANCOVA, MANCOVA, binary logistic regression, and restricted cubic spline models were performed after adjustment for sex, age, and socioeconomic status. **Results**: Higher daily step count was associated with greater cardiorespiratory fitness and with lower symptoms of depression, anxiety, and stress, although the associations with mental health symptoms were weak and not uniform across outcomes. Restricted cubic spline models showed inverse non-linear associations for mental health symptoms, with steeper inverse gradients at lower step-count levels and a tendency to level off at higher volumes, approximately around 9000 steps/day. For cardiorespiratory fitness, the association was positive across the step-count range. Step counts around 7500 steps/day were associated with lower odds of elevated symptoms of depression, anxiety, and stress. **Conclusions**: A higher daily step count was associated with more favorable mental health symptom profiles and greater cardiorespiratory fitness in this sample of university students.

## 1. Introduction

University life represents a critical period in the life course, not only because students are undergoing professional training but also because it is a stage in which unhealthy habits are frequently adopted, including insufficient sleep, poor dietary behaviors, and high alcohol consumption [1,2], along with reduced physical activity, increased sedentary behavior, and impaired physical fitness [3,4]. These behaviors are associated with a higher risk of developing non-communicable and cardiovascular diseases [5,6].

At the same time, mental health problems are highly prevalent among university students, particularly symptoms of depression, anxiety, and stress [7,8]. Evidence suggests that low levels of physical activity and poor physical fitness may contribute to the onset or worsening of these symptoms [9,10]. In this context, physical activity has been shown to be an effective strategy for reducing symptoms of depression, anxiety, and stress in the general population [11], whereas among university students, higher levels of physical activity and lower sedentary behavior have been associated with better mental health outcomes [12,13]. Similarly, substantial evidence indicates that higher levels of cardiorespiratory fitness are associated with lower levels of psychological distress and fewer symptoms of depression and anxiety [11,14,15], suggesting that cardiorespiratory fitness may be a useful indicator of mental health status. In addition, daily step count has gained increasing attention as a simple and objective metric of habitual ambulatory activity, with the advantage of being easily monitored through wearable devices and translated into practical public health messages [16]. From a conceptual perspective, daily step count may reflect the accumulated volume of habitual movement, whereas cardiorespiratory fitness represents a broader physiological attribute influenced by repeated physical activity exposure, individual adaptation, and functional capacity [17,18]. Thus, although related, these constructs are not interchangeable and examining them together may provide a more comprehensive understanding of both behavioral and physiological dimensions of health.

However, important gaps remain in the literature. First, previous studies have largely examined these associations separately, focusing either on the relationship between daily step count and mental health symptoms [19,20,21,22,23] or on the association between daily step count and cardiorespiratory fitness [17,18]. Moreover, studies examining daily step count and mental health have focused predominantly on depression or general psychological symptoms in adult populations, whereas evidence simultaneously addressing depression, anxiety, and stress in university students remains limited [19,20,21,22,23]. As a result, limited evidence is available on how daily step count, cardiorespiratory fitness, and symptoms of depression, anxiety, and stress are jointly related within the same study, particularly in university students. Second, although some evidence exists in the general population [17,18,19,20,21,22,23], university students represent a distinct group characterized by high academic demands, psychosocial stressors, and health-related behaviors that may influence both physical activity patterns and mental health outcomes. Therefore, findings from other populations may not be directly generalizable to this group. This is especially relevant for cardiorespiratory fitness, as it is an important marker of current and future health [17], yet its role has been less consistently incorporated into studies examining the relationship between daily movement behaviors and mental health in university settings [14,15,17,18]. Third, although daily step count is increasingly used as a practical indicator of physical activity [16], the potential dose–response pattern linking daily steps with cardiorespiratory fitness and mental health symptoms in university students remains insufficiently understood. It is still unclear whether these associations are linear or non-linear, and whether meaningful step-count thresholds may exist for mental and physical health outcomes in this population.

From a theoretical standpoint, a higher daily step count could be linked to lower mental health symptom burden through multiple behavioral and physiological pathways, including greater overall movement exposure, reduced sedentary time, improved self-regulation, and better physical conditioning [9,10,11,16]. At the same time, higher daily step counts may be associated with better cardiorespiratory fitness, which in turn has been linked to more favorable psychological health indicators [14,15,17]. However, in the absence of formal mediation analyses, cardiorespiratory fitness should not be interpreted as a demonstrated mediator of the relationship between daily step count and mental health symptoms but rather as a related and relevant health indicator that may help to contextualize these associations.

Given that a higher daily step count is associated with both better mental health and greater cardiorespiratory fitness, examining these variables jointly may provide a more integrated view of health in university students. Nevertheless, evidence integrating these three components in university students remains scarce. Therefore, the aim of the present study was to determine the association between total daily steps, cardiorespiratory fitness, and symptoms of depression, anxiety, and stress in university students, and to explore the potential dose–response pattern of these associations.

## 2. Materials and Methods

### 2.1. Design

An observational, cross-sectional association study was conducted among university students.

### 2.2. Participants

The sample consisted of 120 students from Universidad Autónoma de Chile, Temuco campus, Chile, including 64 women and 56 men, aged 18 to 25 years. Data were collected between September and November 2025.

Participants were recruited through non-probability convenience sampling. Eligible participants were students enrolled at Universidad Autónoma de Chile who voluntarily agreed to participate and provided written informed consent. Exclusion criteria included a medical or self-reported diagnosis of depression, anxiety, or stress, current pharmacological treatment for any of these conditions, and participation in amateur or professional sports in which daily step count could be markedly elevated, in order to avoid overestimating habitual physical activity levels. These exclusion criteria were applied to reduce potential confounding related to clinically diagnosed mental health conditions, psychopharmacological treatment, and atypically high activity patterns; however, they may also limit the representativeness of the sample with respect to the broader university student population.

### 2.3. Procedure

The study invitation was disseminated through informational posters shared via social media and institutional email. Once the sample had been established, assessments were scheduled and carried out in the university’s physical activity laboratory.

The assessment began with the administration of a sociodemographic questionnaire, followed by the mental health questionnaire. Anthropometric measurements were then obtained, and cardiorespiratory fitness was subsequently assessed at the university sports center. Upon completion of the in-person assessment, each participant received a pedometer to record daily step count for seven consecutive days.

The total assessment time was approximately 40 min per participant. All evaluations were conducted by the principal investigator and by graduates in Physical Education who had received prior training, with this method chosen in order to ensure standardized procedures and to appropriately resolve any questions raised by participants during the administration of the instruments. Participants were instructed on the correct use of the device and on how to export the step-count records at the end of the monitoring period. Step-count data were exported directly from the mobile application linked to the Xiaomi Mi Band 9 (Xiaomi Corporation, Beijing, China) and submitted by email to the research team. After receipt, the files were reviewed for completeness of the 7-day monitoring period, consistency of the recorded values, and compatibility with the expected export format before inclusion in the analyses.

### 2.4. Ethical Considerations

The study protocol was approved by the Scientific Ethics Committee of Universidad Autónoma de Chile (CEC N°28-25) on 3 June 2025. All participants provided written informed consent prior to study enrolment. Participation was voluntary, and all collected information was handled confidentially, with participants’ identities protected throughout all stages of the research.

### 2.5. Measures

#### 2.5.1. Daily Step Count

Daily step count was recorded using Xiaomi Mi Band 9 devices. At the end of the in-person assessment, each participant received the device and was instructed to wear it on the non-dominant wrist for seven consecutive days, for at least 12 h per day, under free-living conditions, following standardized instructions provided by the research team. Participants were asked to use the device as regularly as possible throughout the monitoring period and to maintain their usual daily routines. The variable used in the analyses was the average daily step count calculated from the seven-day monitoring period.

Total step records were obtained from a CSV file downloaded by each participant from the Mi Fitness application (Xiaomi Mobile Software Co., Ltd., Beijing, China) and subsequently sent to the research team by email. A valid daily recording was defined as a day with an available step-count value in the exported file. Only participants with seven valid daily recordings were considered eligible for analysis. Therefore, participants with one or more missing days were excluded, and missing data were handled through complete-case analysis without imputation. Step-count data were reviewed before analysis, and no additional exclusions due to outlying values were applied beyond the requirement of complete seven-day records.

#### 2.5.2. Symptoms of Depression, Anxiety, and Stress

Symptoms of depression, anxiety, and stress were assessed using the self-report version of the Depression Anxiety Stress Scales-21 (DASS-21) [24], a 21-item instrument composed of three dimensions, namely depression, anxiety, and stress, with seven items per dimension. Each item is scored on a 4-point Likert scale ranging from 0 (did not apply to me at all) to 3 (applied to me very much or most of the time). The Spanish version, previously validated in Chilean university students, was used [25]. The instrument yields dimension-specific scores for depression, anxiety, and stress, as well as a total score. Higher scores indicate greater symptom severity. Both the dimension-specific scores and the total score were included in the analyses.

#### 2.5.3. Cardiorespiratory Fitness

Cardiorespiratory fitness was assessed using the 20 m shuttle run test [26]. Based on performance achieved during the test, maximal oxygen uptake was estimated using the equation proposed by Léger [26].

#### 2.5.4. Anthropometric Measurements

Body weight was measured using a digital scale (Tanita MC-780U), whereas height was assessed with a Seca stadiometer (model 200, seca GmbH & Co. KG, Hamburg, Germany). Both measurements were obtained in duplicate, and the mean of the two recordings was used for the analyses. Body mass index (BMI) was then calculated from these values and expressed in kg/m^2^.

#### 2.5.5. Covariates

Age, sex, and socioeconomic status were considered as covariates. Socioeconomic status was determined using the Registro Social de Hogares, a system used in Chile to classify household socioeconomic vulnerability. This instrument considers the income of all household members, divided by the number of individuals in the household, and adjusts this information according to factors such as age, disability, or dependency. The result is expressed as a percentile range from 0% to 90%, with lower values indicating greater socioeconomic vulnerability.

### 2.6. Statistical Analysis

The normality of continuous quantitative variables was assessed using the Kolmogorov–Smirnov test and normal probability plots. Study variables were described as mean and standard deviation. Sex differences were examined using the independent-samples Student *t*-test. When significant differences were observed, effect size was estimated using Cohen’s d, interpreted according to the following thresholds: ≥0.2, small; ≥0.5, moderate; and ≥0.8, large [27].

The associations between daily step count, cardiorespiratory fitness, and the DASS-21 dimensions (depression, anxiety, stress, and total score) were estimated using partial correlation coefficients adjusted for sex, age, and socioeconomic status.

Restricted cubic splines were used to examine the non-linearity of the association between daily steps, depression, anxiety, stress and cardiorespiratory fitness. Four knots were set at the 5th, 35th, 65th and 95th percentiles of the daily step distribution within linear regression models [28] adjusted for age, sex and socioeconomic status. Nonlinearity was assessed using the Wald test by testing the null hypothesis that the coefficient of the spline transformation coefficients were equal to zero.

To estimate the association between daily step count and elevated symptoms of depression, anxiety, and stress, binary logistic regression models were fitted. Mental health symptoms were dichotomized at the median (50th percentile) in order to identify participants with relatively elevated symptom levels within this non-clinical university sample and to facilitate their comparison with those presenting lower symptom levels. Daily step count was dichotomized as ≥7500 versus <7500 steps/day (reference category). This cut-point was selected as a pragmatic and evidence-informed threshold, based on both the distribution of step counts in the sample and emerging evidence indicating that substantial physical and mental health benefits are achieved at step counts below the traditional 10,000-step target, with diminishing returns at higher volumes. Therefore, this cut-point should be interpreted as an analytical rather than a clinical threshold. All models were adjusted for sex, age, and socioeconomic status, and the results were reported as odds ratios (ORs) with 95% confidence intervals and *p*-values.

Additionally, daily step count was categorized into tertiles: the first tertile (low level: <8662 steps/day), second tertile (moderate level: 8662–10,855 steps/day), and third tertile (high level: >10,855 steps/day). Analysis of covariance (ANCOVA) models were used to compare crude mean values of cardiorespiratory fitness and total DASS-21 score across tertiles (Model 0), as well as after adjustment for sex, age, and socioeconomic status (Model 1). Differences in crude mean values of depression, anxiety, and stress symptoms across daily step-count tertiles were examined using multivariate analysis of covariance (MANCOVA) (Model 0), with additional adjustment for sex, age, and socioeconomic status (Model 1), and further adjustment for cardiorespiratory fitness (Model 2). Bonferroni correction was applied for post hoc comparisons.

A *p*-value < 0.05 was considered statistically significant. All analyses were performed using IBM SPSS Statistics, version 29 (IBM Corp., Armonk, NY, USA).

## 3. Results

Table 1 presents the characteristics of the sample overall and according to sex. Comparisons by sex showed that men had significantly higher values for weight, height, cardiorespiratory fitness and daily step count, whereas women showed higher values for depression, anxiety, stress, and total DASS-21 score, with significant differences observed for all these variables. No significant sex differences were observed for body mass index or socioeconomic status.

Table 2 presents the partial correlation coefficients between daily step count, cardiorespiratory fitness, depression, anxiety, stress, and total DASS-21 score. All correlations were statistically significant, although their magnitudes ranged from weak to strong. Daily step count showed a moderate positive correlation with cardiorespiratory fitness and weak inverse correlations with depression, anxiety, stress, and total DASS-21 score. In contrast, cardiorespiratory fitness showed weak-to-moderate inverse correlations with the mental health variables, while the correlations among depression, anxiety, stress, and total DASS-21 score were strong and positive.

Table 3 presents mean differences in cardiorespiratory fitness and total DASS-21 score according to daily step-count tertiles. Participants in the highest tertile of daily steps had significantly higher cardiorespiratory fitness and lower total DASS-21 scores than those in the lowest tertile (*p* < 0.05). These differences were evident in the unadjusted model for both variables; however, statistical significance was no longer observed for total DASS-21 score following adjustment for age, sex, and socioeconomic status.

Table 4 indicates that participants in the highest daily step-count tertile had lower symptoms of depression and stress than those in the lowest tertile, with significant differences observed across all adjustment models. Regarding anxiety symptoms, participants in both the second and third tertiles showed significantly lower values than those in the first tertile in the unadjusted model. However, following adjustment for sex, age, and socioeconomic status, statistical significance was maintained only for the comparison between the third and first tertiles. After additional adjustment for cardiorespiratory fitness, the between-tertile differences were attenuated and no specific pairwise differences remained significant, although the overall model remained statistically significant.

**Figure 1:**
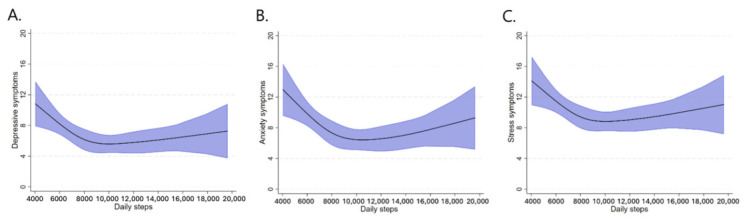
Association between daily step count and symptoms of depression, anxiety, and stress modeled using restricted cubic splines: (**A**) depressive symptoms; (**B**) anxiety symptoms; (**C**) stress symptoms. The model is adjusted for age, sex, and socioeconomic status.

Restricted cubic spline models, adjusted for age, sex, and socioeconomic status, revealed inverse non-linear L-shaped associations between daily step count and symptoms of depression, anxiety, and stress (*p* for non-linearity <0.05). Across all three outcomes, the curves showed a steeper inverse gradient at lower daily step-count levels, particularly below approximately 9000 steps/day. Beyond this range, the curves progressively flattened, indicating that the inverse association between daily step count and mental health symptoms was less pronounced at higher step-count levels (Figure 1).

**Figure 2:**
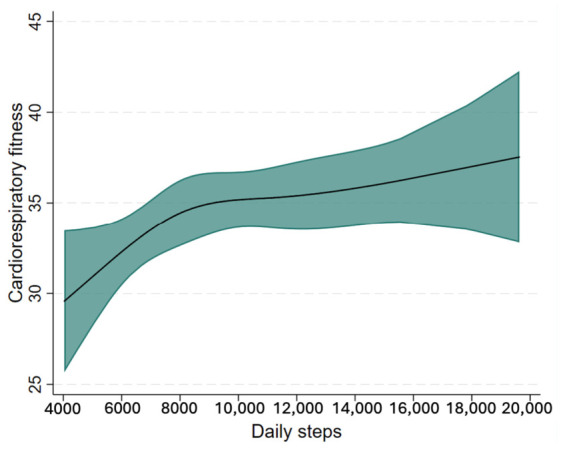
Association between daily step count and cardiorespiratory fitness modeled using restricted cubic splines. The model is adjusted for age, sex, and socioeconomic status.

With respect to the association between daily step count and cardiorespiratory fitness, the restricted cubic spline model, adjusted for age, sex, and socioeconomic status, showed a positive and predominantly linear association (*p* for non-linearity > 0.05). The curve suggested a steeper positive gradient up to approximately 8500 steps/day, after which the slope appeared to gradually attenuate (Figure 2). Overall, higher daily step counts were associated with higher levels of cardiorespiratory fitness across the observed range, although the slope of the association appeared less pronounced at higher step-count levels.

Supplementary Table S1 presents the results of the binary logistic regression model assessing the association between daily step count (≥7500 steps/day) and elevated symptoms of depression, anxiety, and stress (dichotomized at the 50th percentile), adjusted for sex, age, and socioeconomic status. Accumulating ≥7500 steps/day was significantly associated with lower odds of elevated depression (OR = 0.24; 95% CI 0.085–0.674; *p* = 0.007), anxiety (OR = 0.212; 95% CI 0.071–0.633; *p* = 0.005), and stress symptoms (OR = 0.146; 95% CI 0.048–0.445; *p* = 0.001).

## 4. Discussion

The present study aimed to determine the association between total daily step count, cardiorespiratory fitness, and symptoms of depression, anxiety, and stress in university students. The main findings indicate that a higher daily step count was associated with lower symptoms of depression, anxiety, and stress, as well as with greater cardiorespiratory fitness. Specifically, the association between daily step count and mental health symptoms followed a non-linear, inverse L-shaped pattern, characterized by a steeper inverse gradient at lower step-count levels, and a subsequent tendency to level off at approximately 9000 steps/day. This pattern is consistent with recent evidence indicating that greater daily step accumulation is associated with fewer depressive symptoms [19] and suggests that the largest mental health benefits may occur when individuals move from very low levels of daily movement to more moderate step-count ranges. In line with this interpretation, our findings showed that the reduction in symptoms of depression, anxiety, and stress was more pronounced at the lower end of the step-count distribution, after which the association tended to plateau. This supports the idea that even modest increases in daily physical activity may be meaningfully associated with better mental and physical health outcomes [29,30,31,32,33]. Furthermore, the analyses suggest that accumulating at least 7500 steps/day was associated in this sample with lower odds of elevated symptoms of depression, anxiety, and stress. In this context, although a goal of 10,000 steps/day may still be appropriate for individuals with higher activity levels, accumulating around 7000 steps/day has also been linked to clinically relevant health benefits and may represent a more realistic and attainable target for some population groups [32]. Accordingly, the threshold observed in the present study should not be interpreted as a universal prescription, but rather as an empirical reference point that may help contextualize potentially meaningful levels of daily movement. In parallel, daily step count was positively and linearly associated with cardiorespiratory fitness, although with diminishing returns beyond approximately 8500 steps/day. Taken together, these findings indicate that higher levels of daily physical activity may be associated with more favorable both mental health and physical fitness in university students, a population that is particularly exposed to academic stress, behavioral changes, and sedentary lifestyles [34,35].

Our findings are consistent with the growing body of literature challenging the notion that the benefits of physical activity are only meaningful upon reaching the traditional 10,000 steps/day target [29,30,36]. Recent studies have shown that considerably lower step counts are associated with substantial reductions in all-cause mortality, cardiovascular disease, and cardiometabolic risk, with curvilinear rather than linear association patterns [19,29,30,31,32]. This broader pattern has also been supported by recent evidence synthesis on step count and multiple health outcomes, which suggests that the greatest health gains tend to occur when individuals move from very low to moderate levels of daily steps rather than at the highest volumes. In this regard, the greatest benefits tend to be concentrated in the transition from lower to moderate step-count levels, after which the slope of improvement becomes less pronounced [29,30,31,32]. However, these studies are mainly derived from outcomes and populations different from those examined here, so the values observed in the present study should be interpreted as sample-specific patterns rather than definitive thresholds. Thus, although our results should not be interpreted as establishing a universal target, they suggest that in this sample, step counts around 7500 steps/day were associated with lower odds of elevated symptoms of depression, anxiety, and stress, while the spline models indicated that the inverse associations tended to become less pronounced at higher step-count levels. Given the cross-sectional design, these findings do not support causal inferences or prescriptive cut-points but rather provide an empirical reference point for future longitudinal and intervention studies in university students.

The inverse associations observed between daily step count and symptoms of depression, anxiety, and stress are broadly consistent with the previous literature showing beneficial relationships between physical activity and mental health [11,12,19,20,21,22,23,37]. However, it should also be noted that the correlations between daily step count and the DASS-21 dimensions were weak, indicating that although statistically significant, the magnitude of these associations was modest. Several mechanisms have been proposed to explain the relationship between physical activity and mental health, including improvements in affect regulation, self-esteem, sleep quality, neurobiological functioning, and stress buffering [33,34,38,39]. In addition, when walking takes place in outdoor environments or social contexts, further benefits may arise from exposure to natural settings, interpersonal interaction, and reduced sedentary time [40,41]. Although most of the available evidence has focused on depression, growing support also exists for a beneficial association of physical activity with anxiety and perceived stress [39,40,41,42]. By showing a convergent pattern across the three DASS-21 dimensions, our findings support the notion that everyday movement may be related to different domains of mental health in young adults [39,40,41].

Regarding cardiorespiratory fitness, our results showed a positive association with daily step count, with a steeper positive gradient to approximately 8500 steps/day followed by a subsequent flattening of the curve. This finding is consistent with the literature identifying cardiorespiratory fitness as one of the most robust markers of current and future health and recognizing that initial increases in physical activity often translate into relatively greater gains among less active individuals or those with lower baseline fitness [43,44]. In addition, when cardiorespiratory fitness was included in the adjusted models, the associations between daily step-count tertiles and some mental health outcomes were attenuated, particularly for anxiety. However, this pattern should not be interpreted as evidence of mediation or of a specific mechanistic role of cardiorespiratory fitness, since such pathways were not formally tested in the present study. Rather, these results suggest that cardiorespiratory fitness may represent a related physiological factor that helps contextualize the association between habitual movement and mental health symptoms. In addition, this result complements the mental health findings by suggesting that daily step accumulation may have parallel relevance across psychological and physiological domains, thereby reinforcing the utility of step count as a simple and integrative indicator of health in university populations. Nevertheless, the practical implications of these findings should be interpreted cautiously, since the observed associations are cross-sectional and derived from a specific university sample. The relevance of this finding lies in the fact that it broadens the interpretation of the results by showing that increasing daily step count was associated not only with lower mental health symptom scores, but also with a central component of physical and functional health during a stage of life in which habits that may persist into adulthood are being consolidated [43,44].

The findings of the present study should be interpreted considering several limitations. First, the cross-sectional design precludes establishing causal relationships or the temporal direction of the observed associations. Second, although the analyses were adjusted for age, sex, and socioeconomic status, residual confounding by unmeasured variables cannot be ruled out, including sleep quality, dietary habits, academic workload, substance use, pre-existing health conditions, or social support. Third, although step count represents a practical and highly useful measure, it does not fully capture dimensions such as exercise intensity, the context in which movement occurs, or participation in other types of physical activity. Additionally, the interpretation of step-based thresholds should remain cautious, as evidence in this field is still constrained by the limited number of available studies for several outcomes, the scarcity of age-specific analyses, and potential biases at the individual study level, including residual confounding [34]. Nevertheless, the study also has important strengths, including the joint assessment of daily step count, cardiorespiratory fitness, and symptoms of depression, anxiety, and stress; adjustment for relevant sociodemographic covariates; and the application of restricted cubic spline models, which allowed the exploration of non-linear associations and the identification of thresholds of potential clinical and population-level relevance [35,36], although these threshold-like patterns should be interpreted cautiously and not as definitive cut-points.

## 5. Conclusions

In university students, a higher daily step count was associated with lower symptoms of depression, anxiety, and stress, as well as with better cardiorespiratory fitness. The spline analyses suggested a non-linear pattern for mental health outcomes, characterized by larger inverse gradients at lower step-count levels, with the association tending to level off at around 9000 steps/day. In contrast, for cardiorespiratory fitness, a positive and linear association with daily step count was observed, with a steeper gradient up to approximately 8500 steps/day and a subsequent attenuation at higher levels. In this context, achieving around 7500 steps/day was associated, in this sample, with a more favorable mental and physical health profile in this population and should be interpreted as an empirical reference point rather than as a definitive target.

Although these findings may be relevant for health promotion in university settings, they should be interpreted cautiously given the cross-sectional design of the study. The results suggest that favorable mental health and fitness profiles may be observed without necessarily reaching rigid targets such as 10,000 steps/day. From a practical perspective, the findings may help to inform strategies aimed at gradually increasing everyday movement through simple and feasible actions, such as encouraging walking across campus, incorporating active breaks, using devices or mobile applications for step self-monitoring, and developing institutional initiatives designed to reduce sedentary behavior. However, these implications should be regarded as preliminary and should be confirmed by longitudinal and intervention studies before informing specific recommendations. In summary, the central message is that higher daily step accumulation was associated with a more favorable mental health and physical fitness profile in this sample of university students.

## Figures and Tables

**Table 1:** Baseline characteristics of the sample.

	Total (Mean ± SD)	Women (Mean ± SD)	Men (Mean ± SD)	*p* Value	Cohen’s d
	(*n* = 120)	(*n* = 64)	(*n* = 56)		
Weight (kg)	69 ± 12.40	63.34 ± 9.89	75.49 ± 11.92	0.002	1.117
Height (cm)	166.70 ± 9.40	160.08 ± 6.83	174.36 ± 5.33	0.003	2.313
BMI (kg/m^2^)	24.70 ± 3.61	24.75 ± 3.87	24.78 ± 3.3	0.969	-
Socioeconomic Status (%)	45.30 ± 10.96	45.25 ± 11.07	45.35 ± 10.9	0.950	-
Steps per day	10,358.10 ± 4391.30	9532.12 ± 3453.44	11,303.09 ± 5135.31	0.031	0.410
CRF (mL/kg/min)	38.35 ± 7.32	33.54 ± 4.56	43.85 ± 5.87	0.002	1.958
Depression	5.75 ± 4.73	6.94 ± 5.12	4.39 ± 3.85	0.002	−0.557
Anxiety	6.47 ± 5.45	8.34 ± 5.85	4.32 ± 4.03	0.003	−0.791
Stress	8.58 ± 5.32	10.62 ± 5.38	6.25 ± 4.21	0.002	−0.899
Total Score DASS-21	20.8 ± 14.35	25.91 ± 15.09	14.96 ± 10.94	0.002	−0.822

Abbreviations: SD: standard deviation; CRF: cardiorespiratory fitness; DASS-21: Depression, Anxiety and Stress Scale-21 items. Cohen’s d values of 0.2, 0.5, and 0.8 indicate small, intermediate, or strong effect sizes, respectively.

**Table 2:** Partial correlation coefficient between steps per day, cardiorespiratory fitness, depression, anxiety, and stress.

	CRF	Depression	Anxiety	Stress	Total Score DASS-21
Steps per day	0.345 **	−0.086 *	−0.168 *	−0.182 *	−0.153 *
CRF		−0.184 *	−0.401 **	−0.393 **	−0.361 **
Depression			0.787 **	0.716 **	0.894 **
Anxiety				0.845 **	0.952 **
Stress					0.928 **

*p* value = * *p* < 0.05; ** *p* < 0.001. Abbreviations: CRF: cardiorespiratory fitness; DASS-21: Depression, Anxiety and Stress Scale-21 items.

**Table 3:** Analysis of covariance of cardiorespiratory fitness and the total score of depression, anxiety, and stress symptoms by tertiles of steps per day.

		Tertile 1 (T1) <8662	Tertile 2 (T2) 8662–10,855	Tertile 3 (T3) >10,855	*p* Value
	*n*	40	40	40	
CRF (mL/kg/min)	Unadjusted	35.30 ± 5.16 ^T2,T3^	39.51 ± 6.93 ^T1^	40.21 ± 8.61 ^T1^	<0.01
	Adjusted	36 ± 0.78 ^T2,T3^	39.62 ± 0.77 ^T1^	40.31 ± 0.78 ^T1^	<0.01
Total Score DASS-21	Unadjusted	26.40 ± 14.78 ^T3^	19.71 ± 14.30	16.30 ± 12.30 ^T1^	<0.05
	Adjusted	25.51 ± 1.92	19.60 ± 1.91	17.29 ± 1.92	0.06

Data are presented as mean ± standard deviation. Adjusted model is controlling for age, sex, and socioeconomic status. Superscript letter indicates statistical significance (*p* < 0.05) between categories for post hoc tests using the Bonferroni comparisons. Abbreviations: CRF: cardiorespiratory fitness; DASS-21: Depression, Anxiety and Stress Scale-21 items.

**Table 4:** Multivariate analysis of covariance of depression, anxiety, and stress across tertiles of steps per day.

		Tertile 1 (T1) <8662	Tertile 2 (T2) 8662–10,855	Tertile 3 (T3) >10,855	*p* Value
Depression	Unadjusted	7.20 ± 4.85 ^T3^	5.45 ± 4.60	4.60 ± 4.32 ^T1^	<0.05
	Adjusted	7.04 ± 4.75 ^T3^	5.25 ± 4.67	4.46 ± 4.37 ^T1^	<0.01
	Adjusted + CRF	7.08 ± 4.81 ^T3^	5.18 ± 4.61	4.24 ± 4.33 ^T1^	<0.01
Anxiety	Unadjusted	8.60 ± 6.06 ^T2,T3^	5.80 ± 5.41 ^T1^	5 ± 4.15 ^T1^	<0.05
	Adjusted	8.26 ± 7.80 ^T3^	5.76 ± 4.21	5.36 ± 4.1 ^T1^	<0.05
	Adjusted + CRF	8.49 ± 6.93	5.71 ± 4.17	5.08 ± 4.11	<0.05
Stress	Unadjusted	10.60 ± 5.32 ^T3^	8.45 ± 4.96	6.70 ± 5.05 ^T1^	<0.05
	Adjusted	10.27 ± 7.48 ^T3^	8.41 ± 7.46	6.49 ± 5.49 ^T1^	<0.01
	Adjusted + CRF	10.18 ± 7.21 ^T3^	8.39 ± 6.23	6.15 ± 5.12 ^T1^	<0.01

## Data Availability

The data presented in this study are available on request from the corresponding author. The data are not publicly available due to ethical standards.

## Supplementary Materials

The following supporting information can be downloaded at https://www.mdpi.com/article/10.3390/jcm15093191/s1. Table S1: Association between steps per day and symptoms of depression, anxiety, and stress.

## Abbreviations

The following abbreviations are used in this manuscript:

CRF	Cardiorespiratory fitness
DASS-21	Depression Anxiety Stress Scales-21
BMI	Body mass index
OR	Odds ratio
SD	Standard deviation
ANCOVA	Analysis of covariance
MANCOVA	Multivariate analysis of covariance
