# Supplementary material for: Dose–Response Associations Between Daily Step Count, Cardiorespiratory Fitness, and Symptoms of Depression, Anxiety, and Stress in University Students
Source: J Clin Med. 2026 Apr 22;15(9):3191. doi: 10.3390/jcm15093191 (PMC13163769; doi:10.3390/jcm15093191)
Supplement: Supplementary file 1 [file jcm-15-03191-s001.zip › jcm-4260280-supplementary.pdf]

Table S1. Association between steps per day and symptoms of depression, anxiety, and stress

	P value	OR	LL	UL
Depresión	0.0068	0.240	0.085	0.674
Ansiedad	0.0054	0.212	0.071	0.633
Estrés	0.0007	0.146	0.048	0.445

Abbreviations: OR: odds ratio; LL: lower limit of the 95% confidence interval; UL: upper limit of the 95% confidence interval. The reference category for daily steps was <7,500 steps/day. Depression, anxiety, and stress outcomes were dichotomized at the median (50th percentile).
